# *Pseudomonas aeruginosa ttcA* encoding tRNA-thiolating protein requires an iron-sulfur cluster to participate in hydrogen peroxide-mediated stress protection and pathogenicity

**DOI:** 10.1038/s41598-018-30368-y

**Published:** 2018-08-08

**Authors:** Adisak Romsang, Jintana Duang-nkern, Khwannarin Khemsom, Lampet Wongsaroj, Kritsakorn Saninjuk, Mayuree Fuangthong, Paiboon Vattanaviboon, Skorn Mongkolsuk

**Affiliations:** 1 Department of Biotechnology, Faculty of Science, Mahidol University, Bangkok, 10400 Thailand; 2 Center for Emerging Bacterial Infections, Faculty of Science, Mahidol University, Bangkok, 10400 Thailand; 3 Laboratory of Biotechnology, Chulabhorn Research Institute, Bangkok, 10210 Thailand; 4 Molecular Medicine Graduate Program, Faculty of Science, Mahidol University, Bangkok, 10400 Thailand

## Abstract

During the translation process, transfer RNA (tRNA) carries amino acids to ribosomes for protein synthesis. Each codon of mRNA is recognized by a specific tRNA, and enzyme-catalysed modifications to tRNA regulate translation. TtcA is a unique tRNA-thiolating enzyme that requires an iron-sulfur ([Fe-S]) cluster to catalyse thiolation of tRNA. In this study, the physiological functions of a putative *ttcA* in *Pseudomonas aeruginosa*, an opportunistic human pathogen that causes serious problems in hospitals, were characterized. A *P*. *aeruginosa ttcA*-deleted mutant was constructed, and mutant cells were rendered hypersensitive to oxidative stress, such as hydrogen peroxide (H_2_O_2_) treatment. Catalase activity was lower in the *ttcA* mutant, suggesting that this gene plays a role in protecting against oxidative stress. Moreover, the *ttcA* mutant demonstrated attenuated virulence in a *Drosophila melanogaster* host model. Site-directed mutagenesis analysis revealed that the conserved cysteine motifs involved in [Fe-S] cluster ligation were required for TtcA function. Furthermore, *ttcA* expression increased upon H_2_O_2_ exposure, implying that enzyme levels are induced under stress conditions. Overall, the data suggest that *P*. *aeruginosa ttcA* plays a critical role in protecting against oxidative stress via catalase activity and is required for successful bacterial infection of the host.

## Introduction

The ability of pathogenic bacteria to successfully invade a host is largely associated with their ability to rapidly adapt to and overcome host immune systems. Reactive oxygen species (ROS) are reactive molecules and free radicals derived from the incomplete reduction of oxygen. ROS are sequentially produced by the electron transport pathway during aerobic respiration by phagolysosomes in phagocytic cells, which facilitate attacks on invading microbes^1,2^. ROS also play roles in cellular signalling pathways, including apoptosis, necrosis, gene expression, and the activation of cell signalling cascades^3^. An imbalance between the production and removal of ROS (excess ROS) is referred to as oxidative stress, which causes damage to nucleic acids, lipid peroxidation, protein oxidation, enzyme inhibition, and cofactor inactivation^4^. Accordingly, pathogens have evolved mechanisms to protect themselves against host-generated stresses by scavenging excess ROS with cellular enzymes, such as superoxide dismutase (Sod) and catalase (Kat), and the rebuilding of cofactors such as iron-sulfur clusters ([Fe-S]) via the ISC system along with the repair of oxidative damaged proteins via the methionine sulfoxide reductase (Msr) system^5–7^. To attain the highest efficiency and execute successful infection, the complex processes underlying bacterial sensing and responses to stress are controlled by specific mechanisms carried out by various transcriptional regulators^8–10^. For example, OxyR, a LysR-type transcriptional regulator, is a global stress response protein involved in hydrogen peroxide (H_2_O_2_) defence via the activation of genes encoding Kat^11^, while SoxR, a [2Fe-2S] cluster-containing transcription factor, triggers a major response to redox active compounds by activating antioxidant-encoding genes including *sod*^12^. The mechanisms required for adaptive responses to such stresses primarily involve transcriptional controls; however, some bacteria also exhibit adaptive mechanisms for post-transcriptional regulation.

Translational controls in prokaryotes usually involve modifications to tRNA, which is a key molecule for protein synthesis with multiple points of stress-induced regulation^13^. tRNA modifications are catalysed by an enzyme with the potential to influence specific anticodon-codon interactions and regulate translation^14^. A previous study described specific transcripts with particular codon biases encoding stress response proteins that are translationally regulated by dynamic changes in tRNA wobble base modifications^15^. Numerous enzymes have been identified in modification pathways for bacterial tRNAs, such as GidA/MnmE (involved in bacterial virulence in several pathogenic bacteria)^16,17^ and TrmJ (functions in the oxidative stress response in *Pseudomonas aeruginosa*)^18^. *Escherichia coli* TtcA, a 2-thiocytidine tRNA biosynthesis protein, catalyses the post-transcriptional thiolation of cytosine 32 as s^2^C_32_ in some tRNAs^19^. TtcA contains a redox-active and oxygen-sensitive [4Fe-4S] cluster that is chelated by cysteine residues and is absolutely essential for activity^19^. The modified nucleoside s^2^C_32_ has thus far been found in tRNAs from organisms belonging to the Archaeal and Bacterial domains^19^. The TtcA protein family is characterized by the presence of both a PP-loop and a Cys-X-X-Cys motif in the central region of the protein but can be divided into two distinct groups based on the presence and location of additional Cys-X-X-Cys motifs in terminal regions of the protein sequence^20,21^. Mutant analysis in *E*. *coli* showed that both cysteine residues in this central conserved Cys-X-X-Cys motif are required for the formation of s^2^C_32_^19^. The biochemical mechanism of TtcA that catalyses the thiolation of cytosine 32 has been well studied; however, the physiological function of this enzyme has never been reported.

*Pseudomonas aeruginosa* is one of the most common opportunistic human pathogens and causes lethal infections in patients with impaired immune systems or in critical condition. Hospital-acquired infections caused by *P*. *aeruginosa* are increasing with global epidemiology. Expanding our knowledge of the regulatory virulence network in this bacterium will facilitate the identification of potential drug targets. In this study, *P*. *aeruginosa ttcA* encoding TtcA, which contains conserved Cys-X-X-Cys motifs to bind the [Fe-S] cluster, was functionally characterized in response to oxidative stress and was found to play a role in the pathogenicity of this bacterium.

## Results and Discussion

### Identification of *ttcA* in *P*. *aeruginosa*

**Figure 1:**
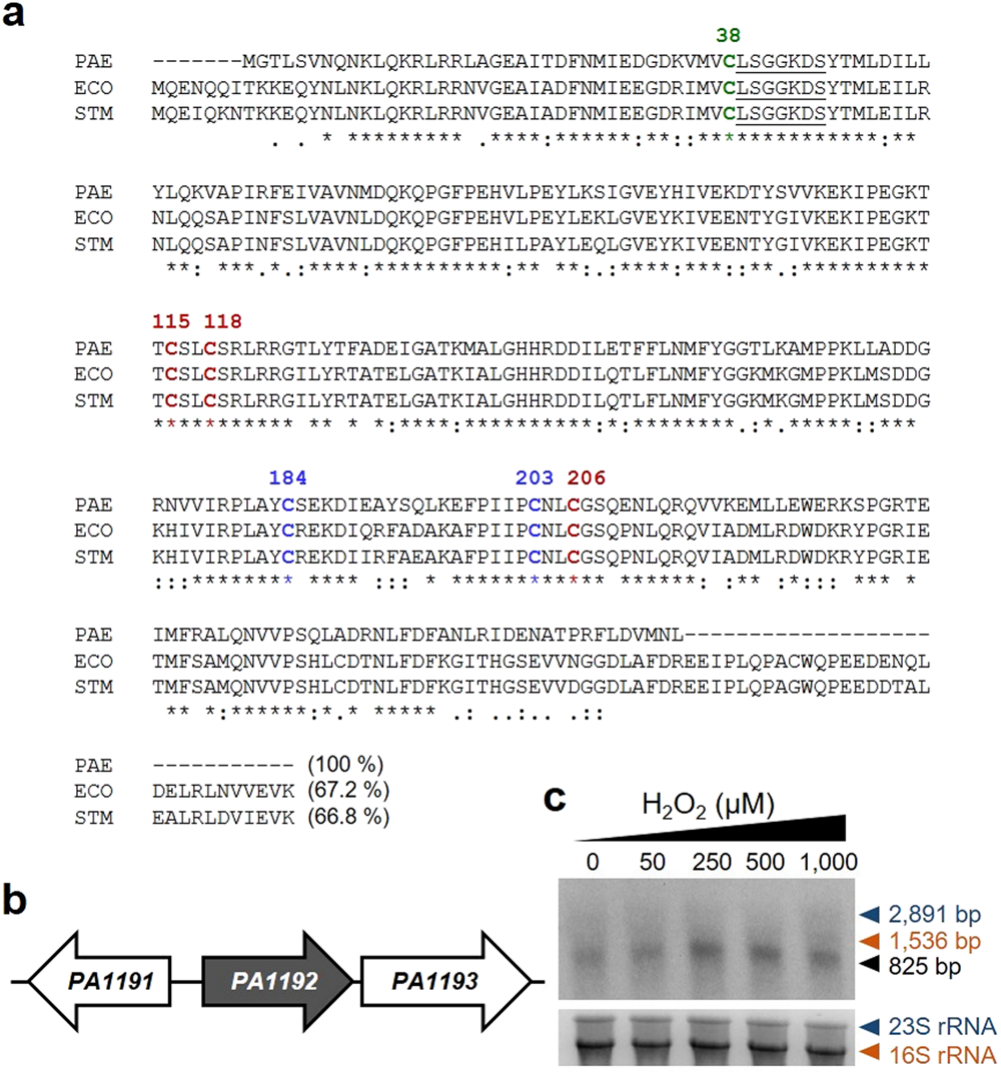
Multiple alignment of *P*. *aeruginosa* TtcA and gene organization around *ttcA*. (**a**) Alignment of TtcA from *P*. *aeruginosa* with TtcA sequences from *Escherichia coli* and *Salmonella enterica* serovar Typhimurium. The alignments were performed using the CLUSTALW algorithm. Underlined and bold letters indicate the amino acids responsible for the PP-loop motif and conserved cysteines in TtcA, respectively. The asterisk, colon, and period symbols indicate identical residues, conserved substitutions, and semi-conserved substitutions, respectively. The numbers on top of the alignments indicate the positions of the amino acids. (**b**) Gene organization of *ttcA* (*PA1192*) in the *P*. *aeruginosa* PAO1 genome. (**c**) Northern analysis of *ttcA* transcript. Total RNA samples were prepared form exponential phase cultures of PAO1 induced with indicated concentrations of H_2_O_2_ and hybridised with ^32^P-labeled *ttcA* specific probe. The gene is transcribed mostly as a monocistronic transcript of approximately 825 bp compared to the size range of ribosomal RNAs. The lower panel shows 16S and 23S ribosomal RNAs at size of 1,536 bp and 2,891 bp, respectively, as a loading control and a size marker.

The *P*. *aeruginosa* PAO1 genome contains the 825-bp open reading frame (ORF) *PA1192*, annotated as a conserved hypothetical gene encoding a protein with high homology to *E*. *coli* TtcA, a tRNA 2-thiocytidine biosynthesis protein^22^. *P*. *aeruginosa PA1192* has a theoretical molecular mass of 31.3 kDa, and its deduced amino acid sequence shares 67.2% and 66.8% sequence identity with TtcA from *Escherichia coli*^19^ and *Salmonella enterica* serovar Typhimurium^21^, respectively (Fig. 1a). No paralogous gene of *PA1192* in the PAO1 genome was found. The TtcA signature motif (LSGGKDS) in the PP-loop family as well as the iron-sulfur cluster binding domains Cys-X-X-Cys (C115-S-L-C118) and Cys-X-X-Cys (C203-N-L-C206) are conserved in *P*. *aeruginosa PA1192* (Fig. 1a). In this study, *P*. *aeruginosa PA1192* was annotated as a putative *ttcA* and further noted as *ttcA*.

*P*. *aeruginosa ttcA* is located 47 bp upstream of *PA1193*, a hypothetical protein (Fig. 1b). Analysis of the transcriptional organization of these genes by Northern blotting and RT-PCR using primers located in the *ttcA* and *PA1193* genes indicated that they are transcribed separately (Fig. 1c). *ttcA* is arranged 102 bp apart from *PA1191*, a hypothetical protein partially containing a putative DnaJ-homologous sequence, in the opposite strand (Fig. 1b).

### Purified TtcA binds an oxidant-sensitive iron-sulfur cluster

**Figure 2:**
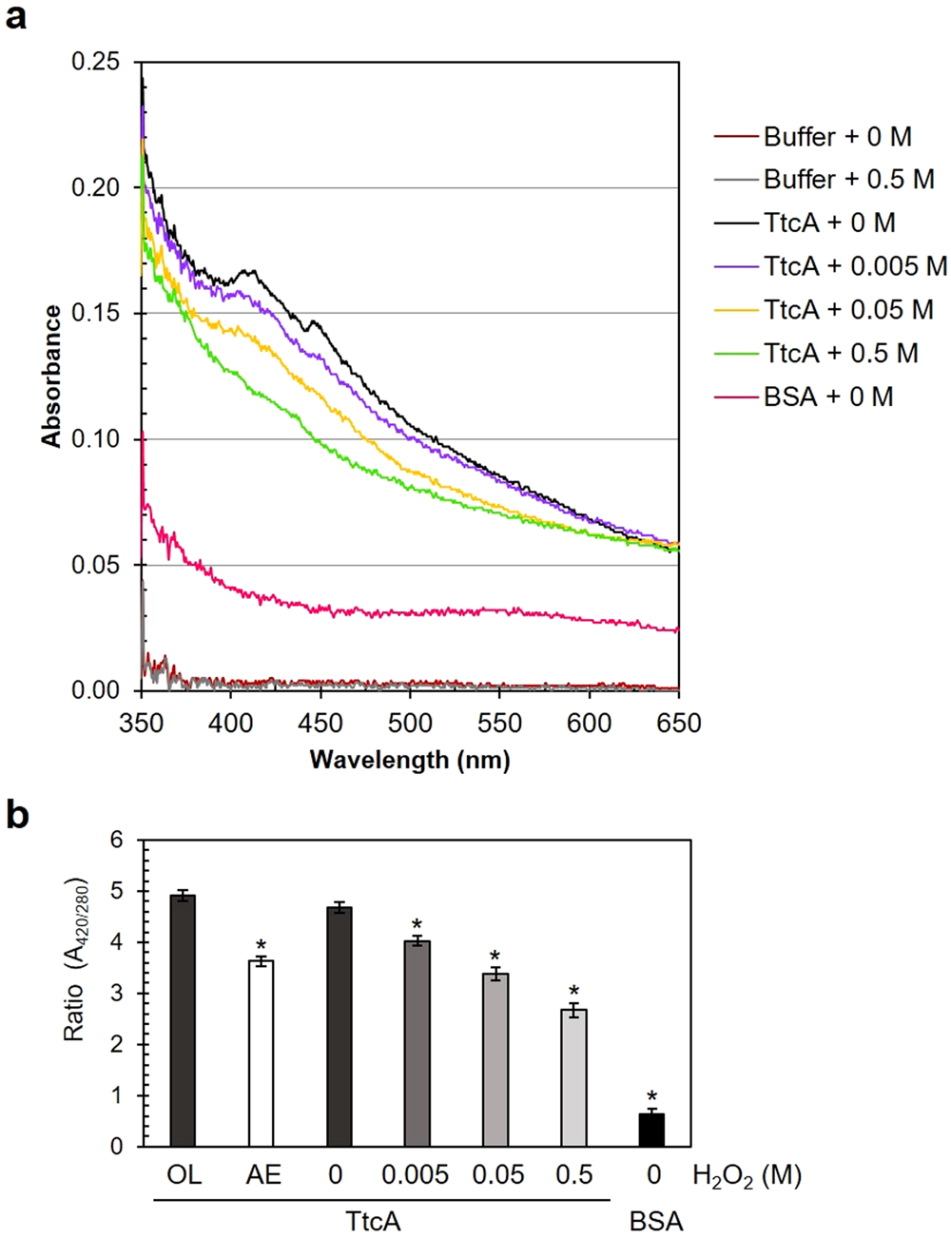
Characterization of iron-sulfur cluster-ligating TtcA in *P*. *aeruginosa*. (**a**) UV-visible absorption spectra of 10 µM purified TtcA protein treated with the indicated concentrations of H_2_O_2_ in 50 mM phosphate buffer were used in the experiments. (**b**) Ratio of the absorbance at 420 nm and 280 nm of TtcA purified under either aerobic (AE) or oxygen-limited (OL) conditions and of TtcA treated with the H_2_O_2_ indicated the relative amount of Fe-S clusters in the protein. BSA (10 µM) was used as the non-[Fe-S] protein control. The asterisk indicates statistical significance (*p* < 0.01) compared with the TtcA without treatment.

To detect iron-sulfur cluster-TtcA ligation, *P*. *aeruginosa* TtcA expression in *Escherichia coli* and TtcA protein purification were performed as described in the Methods. The purified TtcA was then subjected to UV-visible spectroscopy scanning analysis to determine the presence of iron-sulfur clusters. The results in Fig. 2a show significant absorption at 420 nm in the UV-visible spectrum of the purified TtcA, suggesting the presence of a [Fe-S] cluster ligated with the protein, similar to the results of previous studies investigating the characteristics of iron-sulfur cluster proteins^23,24^. This finding was supported by an *in silico* analysis of the iron-sulfur cluster binding domains in the *P*. *aeruginosa* TtcA sequence, which contained two separate Cys-X-X-Cys motifs indicative of [Fe-S] cluster ligation.

Oxidative damage occurs when ROS oxidize an exposed Fe^2+^ atom in the [4Fe-4S] cluster through a metal-based oxidation mechanism, resulting in the ejection of an iron atom from the cluster and subsequent reduction of the cluster to the inactive [3Fe-4S]^+^ oxidation state^25,26^. To determine the effects of H_2_O_2_ on [Fe-S] cluster integrity, purified TtcA was incubated with various concentrations of H_2_O_2_ prior to performing UV-visible spectroscopy. The results showed decreases in TtcA absorbance at 420 nm that were H_2_O_2_ concentration-dependent (Fig. 2a), suggesting that ligation of the [Fe-S] cluster to TtcA provided targets for H_2_O_2_-mediated oxidation (5–50 mM), resulting in the destabilization of iron-sulfur clusters bound to the protein. Treatment of the protein with a high concentration (0.5 M) of H_2_O_2_ led to the total loss of [Fe-S] clusters bound to TtcA, as shown in Fig. 2b. Together with the previously described results, we found that *P*. *aeruginosa* TtcA contains the ROS-sensitive [Fe-S] cluster as its cofactor, similar to TtcA in *E*. *coli*, which contributes to the thiolation of cytosine 32 in tRNA^19^; however, the importance of this cofactor for extended physiological function, particularly under oxidative stress conditions, still needs to be further investigated.

### The Δ*ttcA* mutant shows increased susceptibility to H_2_O_2_ and sodium hypochlorite

**Figure 3:**
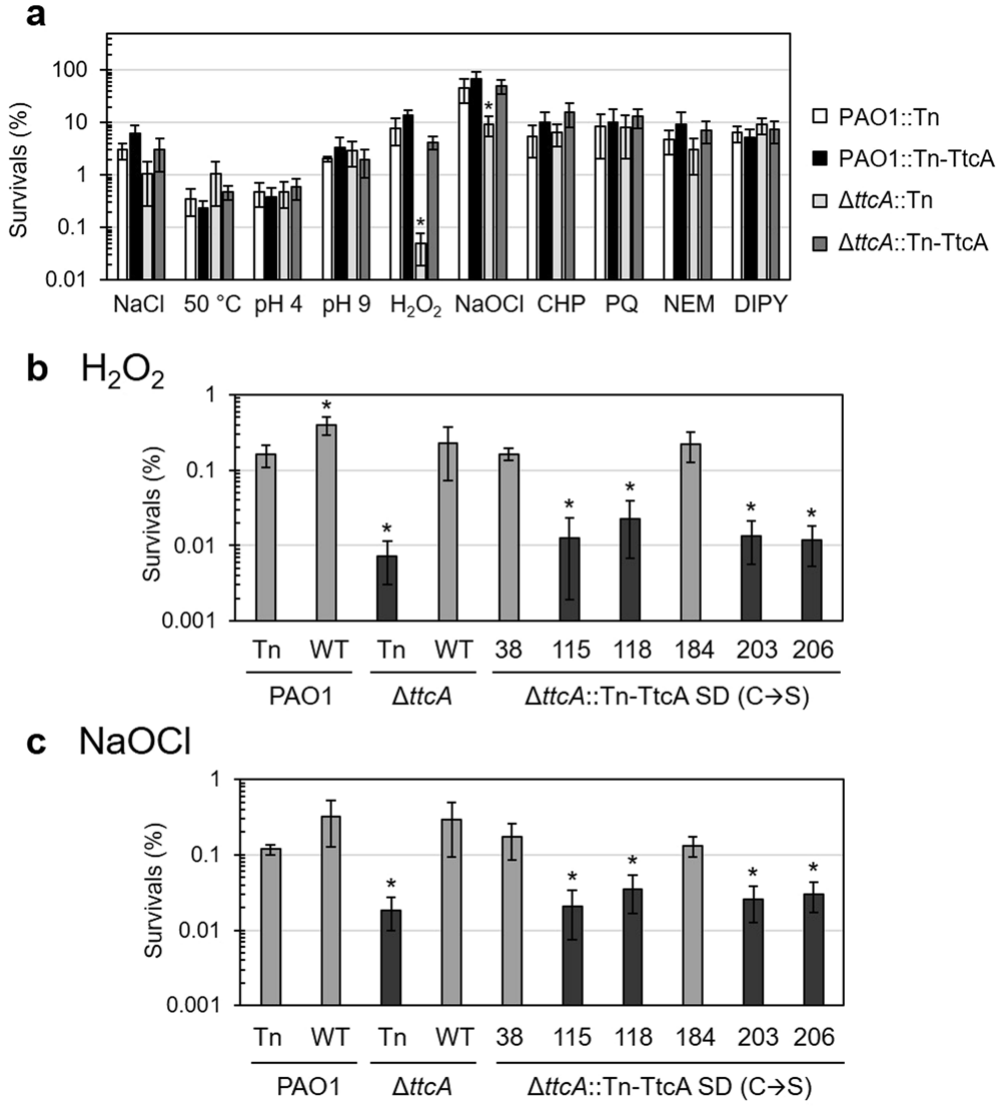
Determination of oxidant resistance levels in *P*. *aeruginosa* strains. (**a**) Oxidant resistance levels in PAO1 and Δ*ttcA* mutants containing the Tn7 insertion in either Tn or Tn-TtcA were determined using plate sensitivity assays. Resistance levels against osmotic (high salt, 5 mM NaCl), heat (high temperature, 50 °C), acidic (pH 4), basic (alkaline, pH 9) and oxidative stresses including H_2_O_2_ (0.5 mM), NaOCl (0.05%), cumene hydroperoxide (CHP, 1.8 M), paraquat (PQ, 200 µM), N-ethylmaleimide (NEM, 0.35 mM) and 2,2′dipyridyl (DIPY, 1.2 mM) were determined using a plate sensitivity assay. (**b**,**c**) show the H_2_O_2_ (120 mM) and NaOCl (0.06%) resistance levels, respectively, of *P*. *aeruginosa* PAO1 and Δ*ttcA* mutants with the Tn7 insertion containing either Tn, Tn-TtcA (WT), or site-directed mutagenic cysteines (C38S, C115S, C118S, C184S, C203S, and C206S), determined using a bacterial killing assay. All data shown are the mean and standard deviation (SD) of the percent survival after incubation for 18 hours from three independent experiments. The asterisk indicates statistical significance (paired t-test, *p* < 0.01) compared with PAO1::Tn treated under the same condition.

To evaluate the physiological function of the *ttcA* in *P*. *aeruginosa* PAO1 against various stresses, a gene deletion mutant (∆*ttcA*) was constructed in PAO1, as described in the Methods. Resistance levels against stresses including: osmotic (high salt, 5 mM NaCl), heat (high temperature, 50 °C), acidic (pH 4), basic (alkaline, pH 9) and oxidative stresses, were determined using a plate sensitivity assay and were compared to that of wild-type PAO1. The oxidants used in this study included: H_2_O_2_, sodium hypochlorite (NaOCl), organic hydroperoxides (cumene hydroperoxide [CHP]), superoxide generators (paraquat [PQ]), a thiol-depleting agent (N-ethylmaleimide [NEM]) and an intracellular iron chelating agent 2,2′-dipyridyl (DIPY). There were no significant differences in the resistance levels of the ∆*ttcA* mutant and wild-type PAO1 against high salt, high temperature, acidic pH, basic pH, organic hydroperoxides, superoxide generators, the thiol-depleting agent and the iron chelator (Fig. 3a). However, the ∆*ttcA* mutant exhibited 50-fold lower resistance to H_2_O_2_ and an 8-fold reduction in the percent survival against NaOCl compared to PAO1 (Fig. 3a). The sensitive phenotype of the ∆*ttcA* mutant against both H_2_O_2_ and NaOCl was complemented by the expression of a single copy of *ttcA* in Tn7 site (Fig. 3a), indicating that TtcA plays an important role in the H_2_O_2_-mediated and NaOCl-derived stress response. In PAO1, the cellular detoxification of H_2_O_2_ primarily depended on catalase activity levels; however, other mechanisms, such as thiol-peroxidase activity (Tpx) and supporting systems, including haem biosynthesis, were also required to achieve fully responsive functionality against H_2_O_2_ in *P*. *aeruginosa*. NaOCl is a bleaching agent that acts as a strong oxidizing agent and can disturb several enzymatic mechanisms, both redox and non-redox, including reactions in tRNA modification processes^27,28^. Moreover, NaOCl has been shown to generate intracellular ROS, which may increase H_2_O_2_ levels and lead to mutant susceptibility^29^. Although our results showed that *P*. *aeruginosa* TtcA particularly affects the oxidative stress response, it may also alter the translational process of other biological pathways since it could function in the translational control.

Furthermore, PAO1 containing an extra copy of functional *ttcA* did not elevate the levels of resistance against these tested oxidants (Fig. 3a), suggesting that other components in the tRNA modification process are required or another detoxification system compensates for oxidant sensitivities. In addition to TtcA in *P*. *aeruginosa* PAO1, TrmJ, another tRNA-modifying enzyme, has also been shown to function in the oxidative stress response of *P*. *aeruginosa* PA14^18^.

### [Fe-S] cluster-ligated cysteine residues are required for the physiological function of TtcA

To assess the important role of [Fe-S] clusters in TtcA-mediated protection against stress conditions, the site-directed mutagenesis of *ttcA* and a complementation assay were performed. Amino acids were changed from cysteine (C) to serine (S) at different positions in the TtcA, including a cysteine next to the PP-loop motif C38; putative cysteines for [Fe-S] cluster ligation at C115, C118 and C206; and other conserved cysteines, C184 and C203, using pUC18-mini-Tn7T-Gm-*ttcA*; then, mutated genes were transformed and integrated into the chromosome of the ∆*ttcA* mutant. A plate sensitivity assay using lethal concentrations of H_2_O_2_ and NaOCl was performed to compare the susceptibility of bacterial growth between the transformed ∆*ttcA* mutants. The results in Fig. 3b show that increased susceptibility to H_2_O_2_ in the ∆*ttcA* mutant was completely restored to wild-type PAO1 levels in ∆*ttcA* mutants containing either the native *ttcA* cassette (WT), the site-directed *ttcA* cassette with C38S, or C184S. However, H_2_O_2_ susceptibility in the ∆*ttcA* mutant containing the site-directed *ttcA* cassette with either C115S, C118S, C203S or C206S demonstrated similar levels as the ∆*ttcA* mutant (Fig. 3b), indicating no phenotypic restoration among these site-directed mutant strains. Therefore, the four cysteines (C115, C118, C203 and C206) were required for fully functional TtcA to play role in the H_2_O_2_-mediated stress response. Moreover, a similar pattern was obtained with the NaOCl complementation assay, as shown in Fig. 3c, indicating that the site-directed *ttcA* cassette containing the cysteine residues (either C115S, C118S, C203S or C206S) was unable to restore NaOCl susceptibility of the ∆*ttcA* mutant to wild-type PAO1 levels, resulting in a sensitivity level similar to the ∆*ttcA* mutant. This suggested that these four cysteines were also required for the TtcA functionality in the NaOCl-mediated stress response. Similar observations regarding the importance of this conserved Cys-X-X-Cys motif in the TtcA protein have been reported for the thiolation of the cytidine in position 32 of tRNA in *S*. Typhimurium^21^ and in *E*. *coli*^19^. Mutation of C219 to alanine in *E*. *coli* TtcA, mapped as C203 of *P*. *aeruginosa* TtcA, exhibited a 50% reduction in tRNA thiolation activity^19^, which was supported by the observed importance of this cysteine residue for the full function of TtcA. However, the exact function of these four cysteine residues in the TtcA, i.e. their possible involvement in the [Fe-S] cluster ligation, is under investigation.

### The Δ*ttcA* mutant exhibits decreased total catalase activity via KatA function

**Figure 4:**
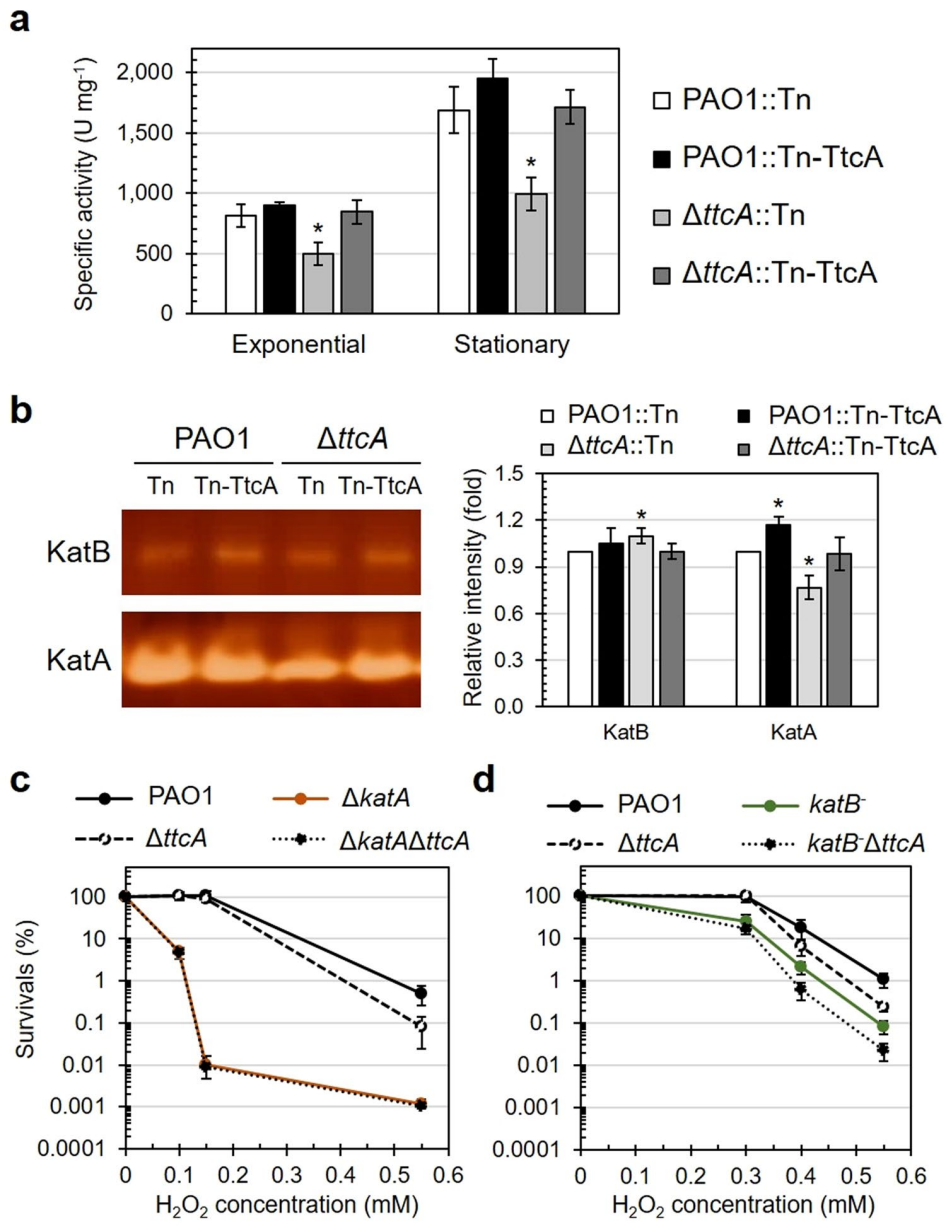
Catalase activity in *P*. *aeruginosa* strains. (**a**) Total intracellular catalase activity was determined in both exponential and stationary cultures of *P*. *aeruginosa* strains. The data shown are the mean and SD of catalase specific activities in each strain from three independent experiments. The asterisk indicates statistical significance (*p* < 0.05) compared with PAO1::Tn at the same growth phase. (**b**) KatA and KatB catalase gel activities were investigated among *P*. *aeruginosa* strains. The band intensity of each tested strain was calculated as the relative intensity (fold change) compared to that of PAO1::Tn, with an asterisk indicating statistical significance (*p* < 0.05). The full-length gel was shown in the Supplementary Fig. S1. H_2_O_2_ resistance levels in the double Δ*katA*Δ*ttcA* (**c**) and *katB*^−^Δ*ttcA* (**d**) mutants compared to that of the PAO1 wild type and the single Δ*ttcA*, Δ*katA*, and *katB*^−^ mutants were determined using plate sensitivity assays and are shown as the mean and SD of the percent survival from three independent experiments.

In several pathogenic bacteria, the cellular detoxification of H_2_O_2_ mainly depends on catalase activity levels. The two major catalases KatA and KatB are responsible for cellular H_2_O_2_ detoxification in *P*. *aeruginosa* PAO1^11,30^. To investigate the involvement of TtcA in the H_2_O_2_-mediated stress response through catalase activity, a total intracellular catalase activity assay was performed in wild-type PAO1 and the ∆*ttcA* mutants. The results showed that total catalase activity in the ∆*ttcA* mutant was 39% and 41% lower than that in wild-type PAO1 under the exponential and stationary phases, respectively, while the ∆*ttcA* mutant harbouring a functional *ttcA* cassette at the Tn7 site showed catalase activity levels similar to that of wild type (Fig. 4a). This result suggested that TtcA is required for full catalase activity in *P*. *aeruginosa* PAO1 under both the exponential and stationary phases.

To determine whether TtcA has roles in KatA or KatB activity, a catalase gel activity assay was performed. The results in Fig. 4b show that KatA activity in the ∆*ttcA* mutant was decreased compared to that in wild-type PAO1, and activity was restored by the expression of the functional *ttcA* cassette at the Tn7 site, as shown in the complemented strain. However, KatB activity levels were slightly increased in the ∆*ttcA* mutant compared to those in wild-type PAO1 (Fig. 4b). These data support the hypothesis that TtcA might have a direct role in KatA activity and may disrupted the H_2_O_2_-responsive pathways. To confirm the contribution of TtcA to KatA activity, double ∆*katA*∆*ttcA* and *katB*^−^∆*ttcA* mutants were constructed and used to determine H_2_O_2_ susceptibility levels in a plate sensitivity assay. If TtcA plays a role in KatA function, the sensitivity of the double mutant should be the same as that of the *katA* mutant. By contrast, if TtcA plays no role in the maintenance of KatA activity, the sensitivity in the double mutant should be additive compared to the single *katA* mutant. The results in Fig. 4c show that the ∆*katA* mutant was more than 1,000-fold more sensitive to H_2_O_2_, while the ∆*ttcA* mutant was approximately 50-fold less sensitive to H_2_O_2_ compared to the sensitivity of wild-type PAO1. Additionally, the double ∆*katA*∆*ttcA* mutant exhibited H_2_O_2_ susceptibility levels similar to the ∆*katA* mutant under a range of H_2_O_2_ concentrations with differing lethality (Fig. 4c), suggesting that TtcA contributes to KatA activity against H_2_O_2_ toxicity. Moreover, the results in Fig. 4d show that the *katB*^−^ mutant was approximately 100-fold more sensitive to H_2_O_2_, while the double *katB*^−^∆*ttcA* mutant exhibited increased H_2_O_2_ susceptibility (20-fold) relative to the *katB*^−^ mutant (Fig. 4d), suggesting that TtcA contributes to H_2_O_2_ resistance primarily through regulation of KatA activity.

### Deletion of *ttcA* causes a change in the expression of genes involved in the oxidative stress response

**Figure 5:**
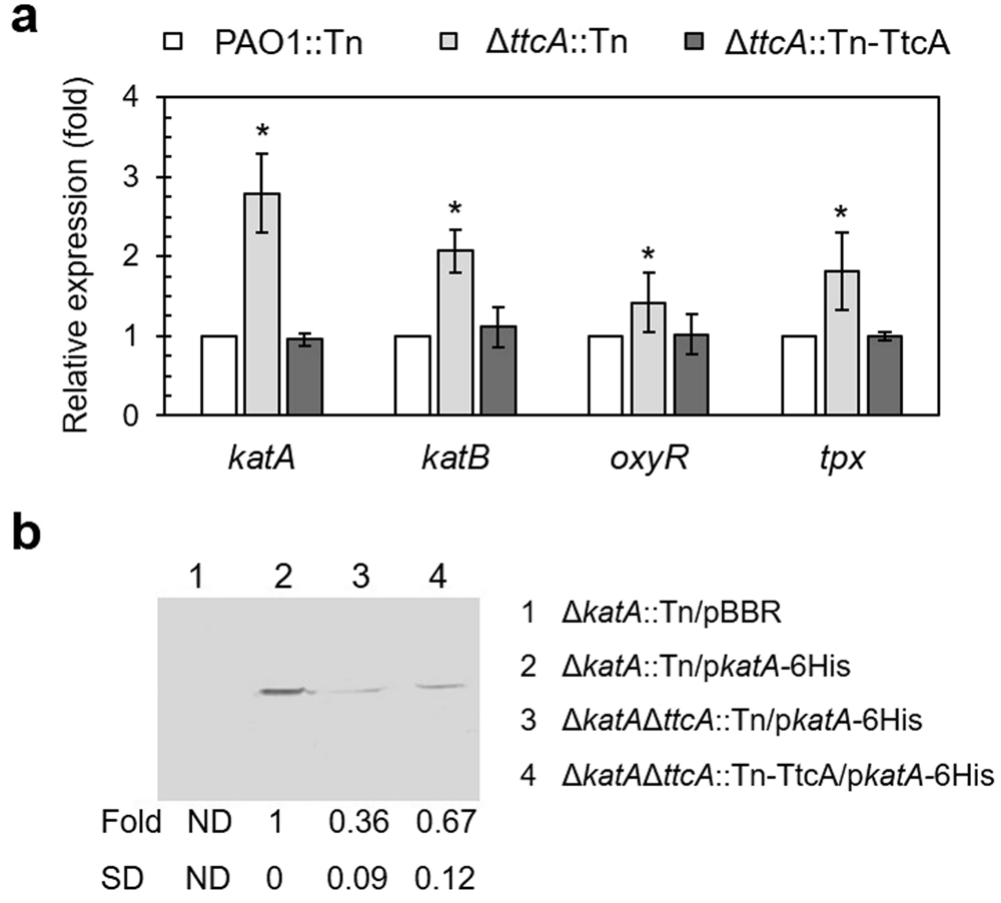
Transcriptional and translational analysis of oxidative stress responsive genes. (**a**) Expression levels of *katA*, *katB*, *oxyR*, and *tpx* in wild-type PAO1 and Δ*ttcA* mutants containing either Tn or Tn-TtcA insertions were determined using real time RT-PCR. Bacterial cultures were grown to the exponential cell phase prior to RNA extraction. Relative expression was analysed using the 16 S rRNA gene as the normalizing gene and expressed as the fold expression relative to wild-type PAO1 levels. The data shown are the mean and SD from three biologically independent experiments. The asterisks indicate statistically significant differences (*p* < 0.01) compared with PAO1 levels. (**b**) Western blot analysis of 6His-KatA levels in *P*. *aeruginosa* strains was determined using a mouse anti-6His antibody. Crude proteins were prepared from an equal amount of *P*. *aeruginosa* culture, and electrophoresis was carried out using 12.5% SDS–PAGE with protein markers. The full-length blot was shown in the Supplementary Fig. S2.

To test whether the deletion of *ttcA* contributed to decreased KatA activity either at the transcriptional level or at the post-transcriptional level, expression analysis of *katA* in the ∆*ttcA* mutant compared to wild-type PAO1 was performed using real time RT-PCR analysis. The results in Fig. 5a show that *katA* expression in the ∆*ttcA* mutant was approximately three-fold higher than that in PAO1 under conditions lacking an oxidant, and increased *katA* expression in the ∆*ttcA* mutant was fully restored to wild-type levels by the extra copy of functional *ttcA* inserted at the Tn7 site. This suggests that decreased KatA activity in the ∆*ttcA* mutant does not result from altered *katA* expression at the transcriptional level; however, it may arise from post-transcriptional control, as it has previously been shown that TtcA has roles in translational control^19^, and decreased KatA activity was observed in this study. To extend our gene expression analysis, the expression profile of genes involved in the oxidative stress response, such as *katB*, *oxyR*, and *tpx*, was determined by performing real time RT-PCR analysis. The results in Fig. 5a show a partial increase in *katB*, *oxyR* and *tpx* expression in the ∆*ttcA* mutant compared to the expression in wild-type PAO1. Furthermore, the expression of *katB* and *tpx* was slightly increased in the ∆*ttcA* mutant under H_2_O_2_ exposure, but there was no significant difference among these strains under NaOCl treatment (see Supplementary Fig. S3). All changes in gene expression in the ∆*ttcA* mutant were restored to wild-type levels by a chromosomal insertion of the extra copy of *ttcA*. This suggested that the ∆*ttcA* mutant caused a defect in H_2_O_2_ detoxification via KatA-mediated mechanisms, leading to a global change in gene expression, including *katB*, *oxyR* and *tpx* expression, in response to H_2_O_2_-mediated oxidative stress. This result supports the previous observation in Fig. 4b that KatB activity was slightly increased in the ∆*ttcA* mutant compared to the activity in wild-type PAO1. However, the real time RT-PCR analysis could reflect an effect on both transcriptional alteration and the stability of mRNAs.

Furthermore, to observe the translational efficiency of the *katA* transcript, Western blot analysis was performed using an ectopic 6His-tagged *katA* expression vector on the ∆*katA* mutant background to compare the native *ttcA* (∆*katA*) and *ttcA* deletion (∆*katA*∆*ttcA*) strains. The results shown in Fig. 5b indicate that the relative amounts of 6His-KatA in the *ttcA* deletion mutant (∆*katA*∆*ttcA*/p*katA*-6His, 36%) were dramatically lower than those in the native *ttcA* mutant (∆*katA*/p*katA*-6His, 100%) and were partially restored by the extra copy of *ttcA* under Tn7-mediated expression (∆*katA*∆*ttcA*/p*katA*-6His, 67%). These results suggested a defect in the translational efficiency of 6His-tagged *katA* expression in the absence of functional *ttcA* and indicated that *ttcA* plays roles in the oxidative stress response at the post-transcriptional level via KatA activity, and the disruption of functional *ttcA* alters the global expression profile of genes involved in oxidative stress management, including induction of *katB* and expression of *tpx*. Western analysis showed only the steady state level of KatA in the cell and therefore could not exclude an effect on KatA degradation.

### The Δ*ttcA* mutant shows attenuated virulence in a *Drosophila* host model

**Figure 6:**
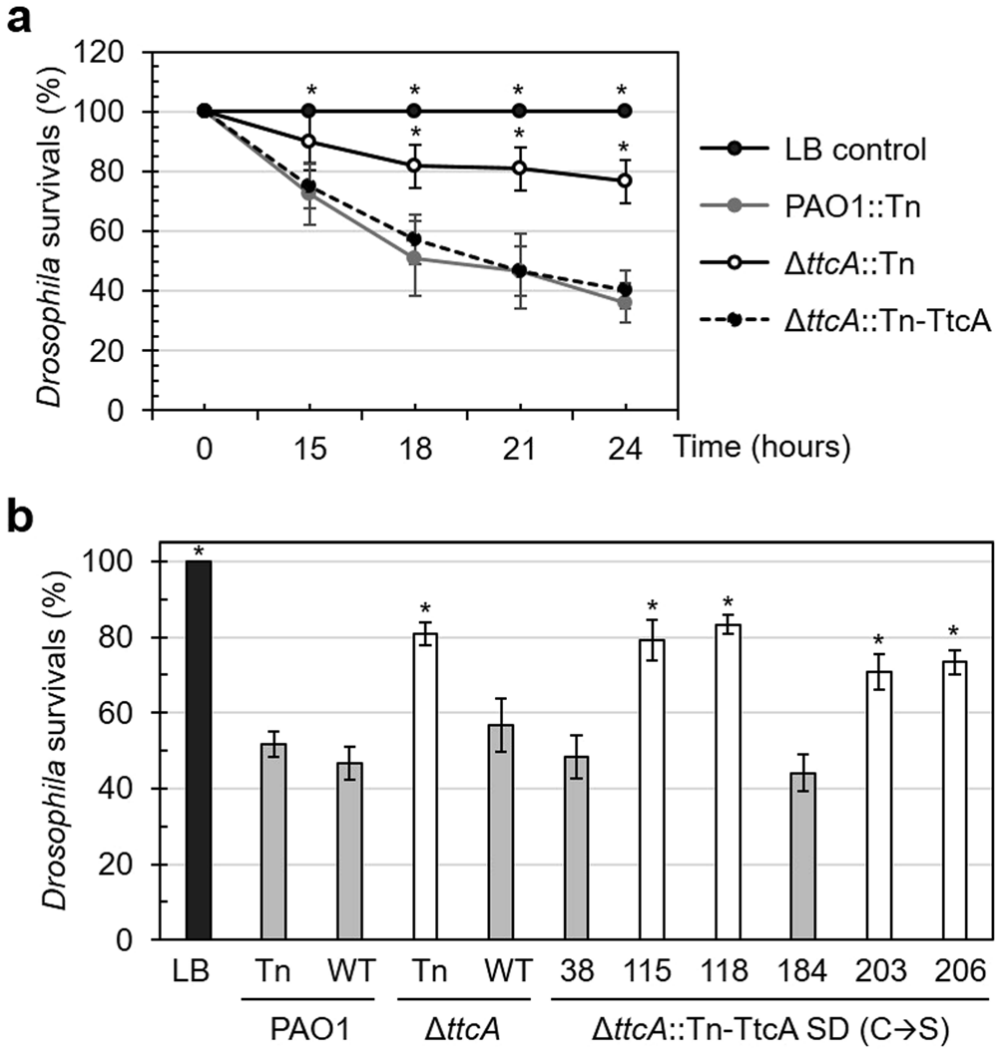
Virulence of *P*. *aeruginosa* strains. (**a**) Virulence of PAO1 and Δ*ttcA* mutants containing the Tn7 insertion in either Tn or Tn-TtcA was determined using the *Drosophila melanogaster* feeding method. The percent fly survival was scored at indicated time points of infection after co-incubation. (**b**) The virulence of PAO1 and Δ*ttcA* mutants containing the Tn7 insertion in either Tn (control), Tn-TtcA (WT), or site-directed mutagenic cysteines in Tn-TtcA (C38S, C115S, C118S, C184S, C203S, and C206S) was determined in the *D*. *melanogaster* feeding assay, and the percent fly survival was scored after co-incubation for 15 hours. The data presented are the mean of three independent experiments, and the error bars in all graphs represent the SD of the mean. Differences in all graphs were statistically evaluated and found to be significant (One-way ANOVA, *p* < 0.01).

The full function of KatA is required for bacterial virulence in several model host systems, as shown in previous studies^31^, and TtcA has been shown to respond to oxidative stress via KatA activity; therefore, the contribution of *ttcA* to the bacterial pathogenicity of *P*. *aeruginosa* was evaluated using *Drosophila melanogaster* as a pathogen-host model. As shown in Fig. 6a, feeding the flies with cultured PAO1 resulted in 50.8 ± 12.5% (after incubation for 15 hours) and 36.0 ± 6.7% (after incubation for 24 hours) fly survival compared with 100 ± 0% (at both time points) fly survival when LB medium was fed to the flies as a negative control. Feeding the flies with Δ*ttcA* mutants resulted in 1.6-fold and 2.1-fold increases in fly survival (81.7 ± 7.4% and 76.7 ± 7.2% after incubation for 15 and 24 hours, respectively) compared with feeding with PAO1 (Fig. 6a). Thus, *ttcA* deletion attenuated the virulence of *P*. *aeruginosa* PAO1 in the tested model (*p* < 0.01). The attenuated virulence phenotype of the Δ*ttcA* mutant was restored in a Δ*ttcA* mutant expressing a functional copy of *ttcA* (57.2 ± 8.2% and 40.5 ± 6.3% fly survival after incubation for 15 and 24 hours, respectively). The attenuated virulence phenotype was consistent with H_2_O_2_ sensitivity levels in the Δ*ttcA* mutant (Fig. 3a). In several plant and animal pathogenic bacteria, defects in peroxide detoxification or repair systems, such as knockout of catalase, methionine sulfoxide reductase or iron-sulfur cluster regulator genes, render the mutant strains attenuated for virulence in model hosts^7,31–33^. Hydrogen peroxide is one of the key components of innate immunity generated by host cells to eradicate invading microbes. In human hosts, H_2_O_2_ is produced within the phagolysosomes of phagocytic cells to kill engulfed pathogens^2^. Thus, defective protection against H_2_O_2_ toxicity in bacteria would reduce survival within the host. Hence, the attenuated phenotype may result from the reduced ability of the Δ*ttcA* mutant to mitigate exposure to H_2_O_2_ during host interactions.

Moreover, the four cysteine residues in TtcA were required for fully functionality in the oxidative stress response via catalase activity. To investigate the requirement of these TtcA cysteine residues in bacterial virulence, complementation with the site-directed *ttcA* mutants was evaluated in a *Drosophila* feeding assay. The results shown in Fig. 6b are similarly to those in Fig. 6a, indicating that feeding the flies with cultured PAO1 either with or without Tn7-mediated insertion of a *ttcA* expression cassette and incubation for 18 hours resulted in approximately 50% fly survival; however, feeding with Δ*ttcA* mutants resulted in an approximately two-fold increase in fly survival. Feeding with the Δ*ttcA*::Tn-TtcA mutant resulted in a fly survival level similar to that of the PAO1 strain (Fig. 6b). Substitution of these particular cysteine residues, either C115, C118, C203, or C206, with serine in the functional *ttcA* expression cassette and insertion into the Δ*ttcA* mutant chromosome did not restore fly survival levels, while replacing one of the other conserved cysteines (either C38 or C184) in TtcA caused the phenotypic restoration of fly survival to wild-type PAO1 levels (Fig. 6b). This indicated that these four particular cysteine residues, including putative amino acids for iron-sulfur cluster ligation (C115, C118 and C206), were required for the complete functionality of TtcA in bacterial pathogenicity. Several *P*. *aeruginosa* genes involved in iron-sulfur cluster biogenesis, including IscR, have been shown to play a role in bacterial virulence, which may correlate with TtcA function in the H_2_O_2_-mediated oxidative stress response through catalase activity.

### *ttcA* expression is increased in response to H_2_O_2_ and NaOCl exposure

**Figure 7:**
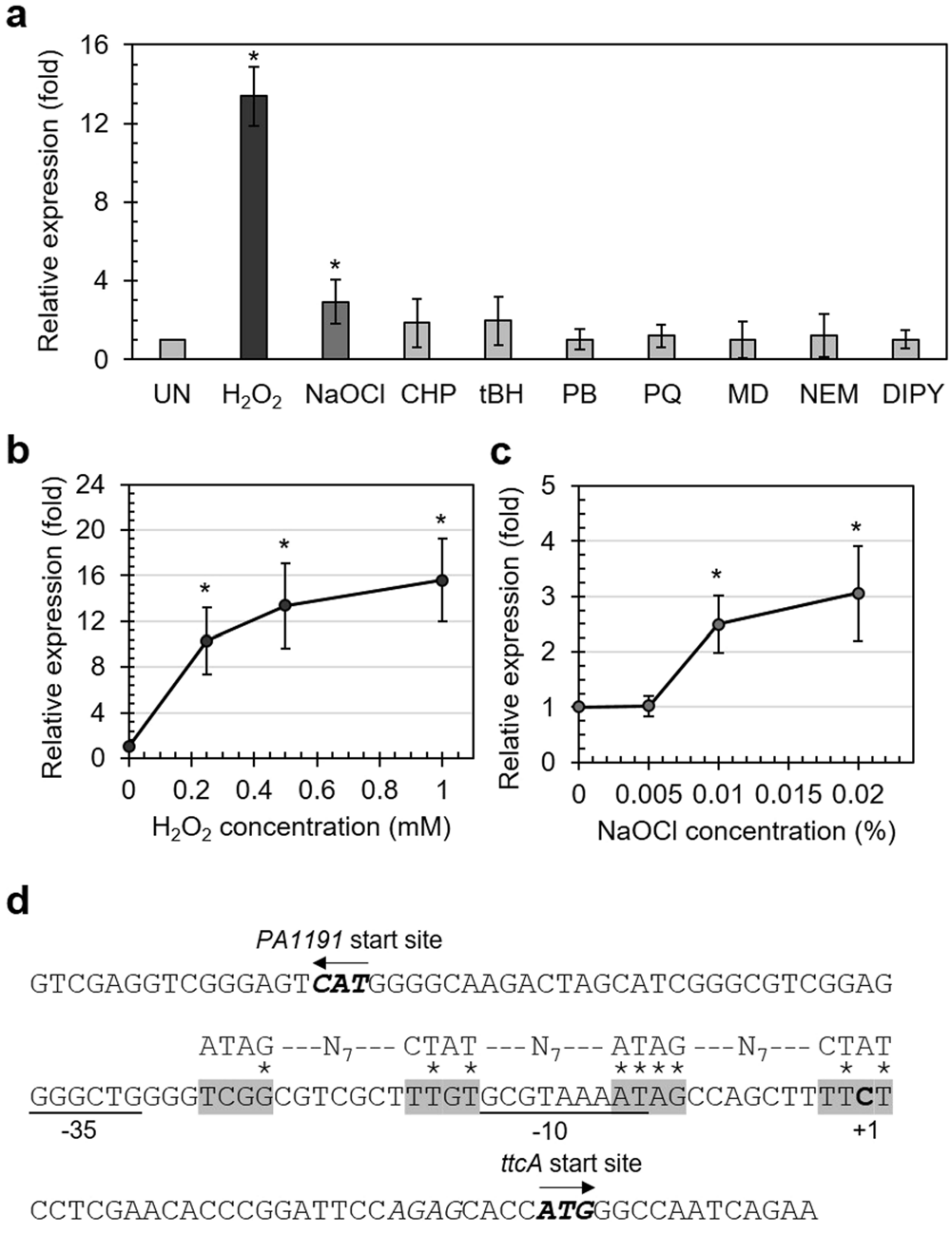
Expression and promoter analyses of *ttcA* in *P*. *aeruginosa* strains. (**a**) Expression levels of *ttcA* under oxidant exposure were determined using real time RT-PCR. Cultures of *P*. *aeruginosa* PAO1 were subjected to various stress conditions, including 0.5 mM H_2_O_2_, 0.02% NaOCl, 0.5 mM cumene hydroperoxide (CHP), 0.5 mM *t*-butyl hydroperoxide (tBH), 0.5 mM plumbagin (PB), 0.5 mM paraquat (PQ), 0.5 mM menadione (MD), 0.1 mM N-ethylmaleimide (NEM) or 1 mM 2,2′-dipyridyl (DIPY) for 15 minutes prior to RNA preparation for real time RT-PCR analysis. Expression levels of *ttcA* in PAO1 under the indicated concentrations of H_2_O_2_ (**b**) and NaOCl (**c**) were determined as in previous experiments. Relative expression and data interpretation were performed as described in previous experiments. (**d**) Nucleotide sequence showing the *ttcA* promoter structure. The putative −10 and −35 promoter elements are indicated as underlined text, and the +1 transcription start site (obtained from the 5′ rapid amplification of cDNA ends (RACE) results) and the ATG translation start site are bolded. The box shaded grey represents the putative OxyR binding site from computational analysis.

Adaptive gene expression is a key component of bacterial defence against environmental stresses. The expression of many genes involved in oxidative stress protection and repair processes is frequently induced by exposure to oxidants^7,34–36^. The expression patterns of *ttcA* in PAO1 cultivated under inducing concentrations of various oxidants were determined using real time RT-PCR. The results illustrated that exposure of PAO1 to organic hydroperoxides, superoxide anion-generating agents, a thiol-chelating agent and an iron-chelating agent did not induce *ttcA* expression (Fig. 7a). By contrast, H_2_O_2_ and NaOCl treatment of PAO1 highly induced *ttcA* expression by 13.4 ± 1.5-fold and 2.9 ± 1.1-fold, respectively (Fig. 7a). The induction of *ttcA* expression by H_2_O_2_ and NaOCl treatment correlated with physiological analysis indicating that TtcA contributes to protection against H_2_O_2_ and NaOCl.

To extend the range of the oxidant-induced gene expression profile, several concentrations of oxidants were applied to bacterial cultures and analysed by real time RT-PCR. The results in Fig. 7b showed that PAO1 cultures were induced with H_2_O_2_ at concentrations ranging from 0.2 mM to 1 mM and in a dose-dependent manner, which was similar to the gene expression profile obtained for genes in the OxyR regulon, including *katA*, *katB*, *ahpB* and *ahpCF*^11,30^. This hinted at the possibility that *ttcA* expression is regulated by OxyR, the global transcriptional regulator responding to H_2_O_2_. Moreover, extending the concentration range for NaOCl treatment from 0.001% to 0.02% showed that *ttcA* expression was not significantly altered under 0.005% NaOCl exposure compared to that in untreated PAO1 (Fig. 7c). This suggested that, unlike H_2_O_2_ induction, induction in response to high concentrations of NaOCl (including a sublethal dose of 0.02%), which slightly induced *ttcA* expression (2.5-fold), may arise from NaOCl reactions generating oxidative stress and probably did not arise via direct NaOCl reactions with the regulator. The NaOCl induction mechanism is under further investigation.

### OxyR modulates the expression of *ttcA* to control catalase activity under stress exposure

**Figure 8:**
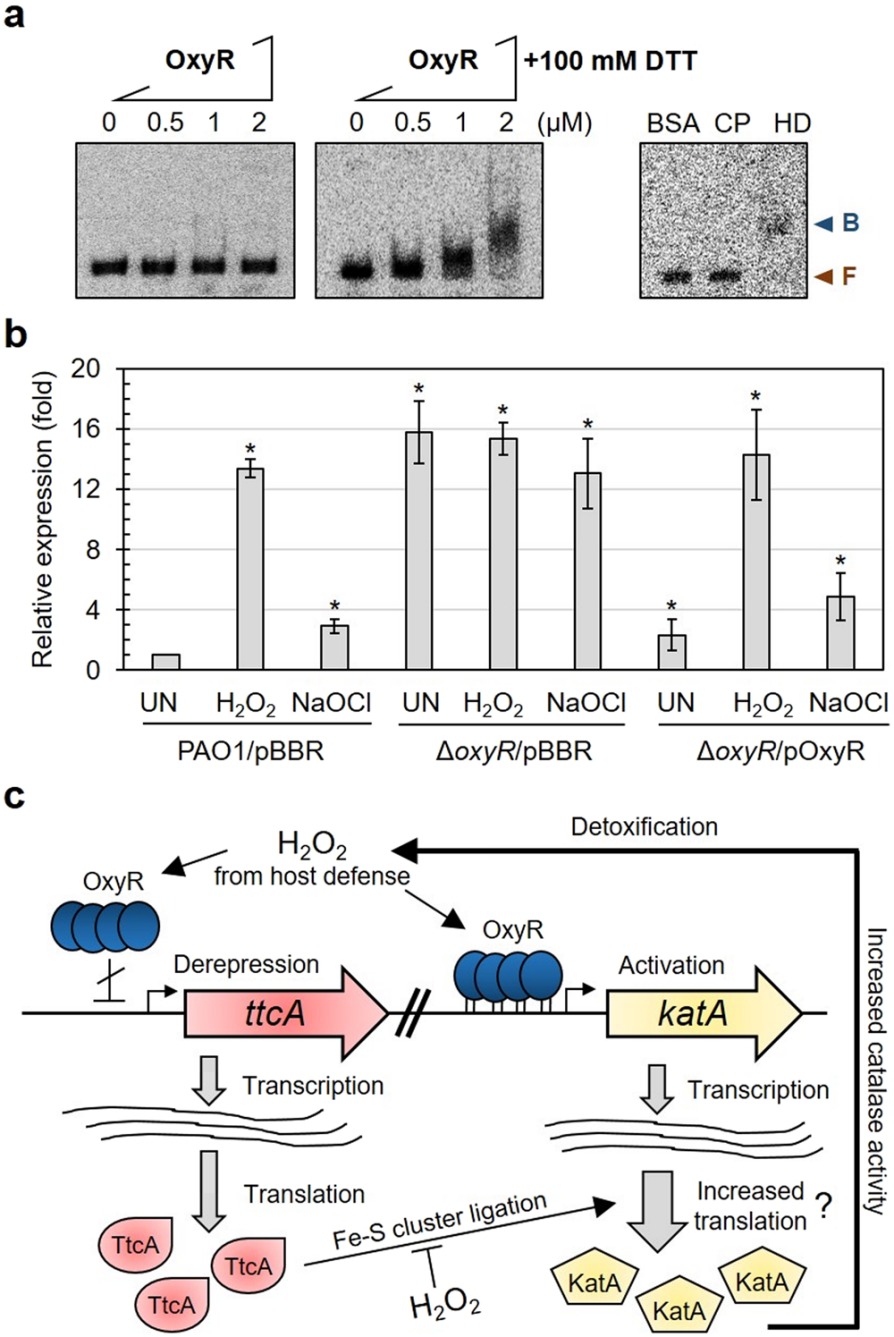
OxyR directly regulates *ttcA* expression. (**a**) Electrophoretic mobility shift assay was performed using ^32^P-labeled *ttcA* promoter fragment and increasing concentrations of purified OxyR in the presence of 100 mM DTT. F and B indicate free and bound probes, respectively. The results from three replications and the full-length gel images were shown in the Supplementary Fig. S4. (**b**) Expression levels of *ttcA* in wild-type PAO1 (PAO1/pBBR), the Δ*oxyR* mutant (Δ*oxyR*/pBBR) and the complemented mutant (Δ*oxyR*/pOxyR) grown under uninduced, 0.5 mM H_2_O_2_, or 0.02% NaOCl induced conditions were investigated using real time RT-PCR and analysed as described in previous experiments. The asterisks indicate statistically significant differences (*p* < 0.01) compared with uninduced conditions. (**c**) Proposed model of OxyR-regulated *katA* and *ttcA* expression under oxidative stress conditions. *P*. *aeruginosa* OxyR upregulates *katA* and *ttcA* expression to increase catalase activities in response to H_2_O_2_ generated by host defence mechanisms. The four cysteine residues required for fully functional TtcA activity to have a role in the oxidative stress response via KatA activity and facilitates bacterial survival during infection.

*ttcA* promoter analysis was performed and physically mapped *in silico*, and the results are presented in Fig. 7d. *ttcA* is located next to *PA1191* with a 102-bp intergenic region. To characterize the *ttcA* promoter, putative +1 sites were investigated using 5′ RACE. The +1 site of *ttcA* was mapped to a cytosine located 28 bp upstream of its translational ATG start codon (Fig. 7d). Two sequences (GGGCTG and GCGTAAAAT, separated by 18 bp) that resembled the *E*. *coli* ơ^70^-35 and -10 promoter motifs were identified. Given the limited intergenic space and a putative promoter sequence analysis, the *ttcA* and *PA1191* promoter motifs might overlap with each other. The canonical OxyR promoter recognition sequence was previously proposed to be ATAG-N7-CTAT-N7-ATAG-N7-CTAT^11^. We mapped the *P*. *aeruginosa ttcA* promoter region and found an upstream sequence (TCGGcgtcgctTTGTgcgtaaaATAGccagcttTTCT) that matched 56% (9 of 16 bases) of the OxyR promoter recognition sequence; therefore, we considered this sequence a putative OxyR binding domain of *ttcA* (Fig. 7d). This putative binding domain overlapped the -10 promoter region, implying an OxyR derepression mechanism for *ttcA* expression in response to oxidative stress. OxyR is a member of the LysR family of transcription regulators, which often use extended palindromic DNA sequences as binding boxes to modulate target gene expression, and diverse consensus sequences for OxyR binding boxes in target gene promoters have been proposed^30^. Direct binding of OxyR to the *ttcA* promoter was determined by electrophoresis mobility shift assay (EMSA). The results in the Fig. 8a demonstrated that purified OxyR binds specifically to the putative *ttcA* promoter in the presence of the reducing agent, dithiothreitol (DTT), suggesting that reduced OxyR is required for the *ttcA* promoter binding. These results support the hypothesis that OxyR directly binds to the *ttcA* promoter and may regulate *ttcA* expression.

To assess whether OxyR regulated the induction of *ttcA* expression upon exposure to oxidative stress, *ttcA* expression levels were examined in an Δ*oxyR* mutant (Δ*oxyR*/pBBR) and a complemented Δ*oxyR*/pBBR-OxyR strain using real time RT-PCR. *oxyR* mutant strains were constructed in PAO1 as described in the Methods. The results showed that under uninduced conditions, the expression of *ttcA* in the Δ*oxyR* mutant was approximately 15-fold higher than *ttcA* levels in PAO1, with *p* < 0.05 (Fig. 8b). H_2_O_2_ and NaOCl treatments did further not enhance the expression of *ttcA* in the Δ*oxyR* mutant. The expression of *oxyR* from the pBBR1MCS-4 vector in the mutant led to the repression of *ttcA* expression to levels similar to those observed in PAO1 (Fig. 8b). Furthermore, the oxidant-induced expression of *ttcA* expression in the complemented strain was restored to wild-type levels (Fig. 8b). These data strongly suggest that OxyR is a transcriptional repressor of *ttcA* expression. Thus, reduced OxyR likely functions as a transcriptional repressor of *ttcA* expression in the absence of the inducers H_2_O_2_ and NaOCl. However, due to the presence of oxidants, oxidized OxyR either activates or derepresses *ttcA* expression, leading to upregulated *ttcA* expression, increased catalase activity, and increased resistance to H_2_O_2_ and NaOCl. OxyR controls a core regulon of oxidative stress defensive genes and other genes involved in the regulation of iron homeostasis, quorum-sensing, protein synthesis and tRNA modification^37,38^. Our results indicate that OxyR is involved in oxidative stress defence through diverse paths of control against H_2_O_2_ as well as NaOCl.

## Conclusion

In this study, the physiological role of tRNA modification through Fe-S cluster-ligated TtcA in the pathogenic bacterium *P*. *aeruginosa* is presented. Under either H_2_O_2_- or NaOCl-mediated stress, direct transcriptional regulation through *kat* gene expression may not be sufficient to control cellular catalase activity, and translational control through tRNA modification is required. Herein, we proposed an additional model (Fig. 8c) involving OxyR regulation to control catalase activity via both direct transcription and indirect translation of TtcA under oxidative stress conditions. During bacterial infection, *P*. *aeruginosa* OxyR upregulates *katA*^39^ and derepresses *ttcA* expression to increase catalase activity in response to H_2_O_2_ generated via host defence mechanisms. It is possible that the consequence of *ttcA* inactivation is a malfunction in tRNA thiolation, which may affect the efficiency of translation, leading to low cellular levels of KatA and the impairment of the oxidative stress response as well as the attenuation of virulence. Although, extended exposure to H_2_O_2_ may cause the disruption of Fe-S cluster-ligating enzymes including TtcA, the low cellular level of active Fe-S clusters would allow the IscR to turn on the ISC system for rebuilding the Fe-S cluster^6^ and to increase the *tpx* expression in order to detoxify the H_2_O_2_^36^. Together with four essential cysteine residues, including putative [Fe-S] cluster coordinators, TtcA has been shown to play an important role in the oxidative stress response and to facilitate bacterial survival during infection of the host, which emphasizes the critical role of the intracellular function of iron-sulfur cluster biogenesis and tRNA modification via IscR and OxyR regulation to mitigate oxidative stress and promote bacterial pathogenicity.

## Methods

### Bacterial strains, plasmids and growth conditions

Both *E*. *coli* and *P*. *aeruginosa* (PAO1, ATCC15692) strains were aerobically cultivated in Lysogeny broth (LB from BD Difco, USA) at 37 °C unless otherwise stated. Exponential phase cells (OD_600_ of 0.5) were used in all experiments. All plasmids used in this study are listed in Supplementary Table S1.

### Molecular techniques

General molecular techniques were performed according to standard protocols^40^. Transformation into *P*. *aeruginosa* strains was carried out using electroporation as previously described^41^. The oligonucleotide primers used in this study are listed in Supplementary Table S2.

### Northern blot analysis

Total RNA isolation, gel electrophoresis, blotting, and hybridization were performed as previously described^35^. For analysis of *ttcA* expression, 20 µg of purified total RNA was loaded into the gel. Radioactively labeled probes were prepared using [α-^32^P]dCTP and a DNA labeling bead (Amersham, GE Healthcare). A 247-bp fragment of the *ttcA* coding region used as a gene-specific probe was amplified from pBBR-TtcA using primers BT4675 and BT4676.

### Construction of *P*. *aeruginosa* Δ*ttcA* mutants

The *ttcA* deletion mutant was constructed using homologous recombination with an unmarked Cre-*loxP* system as previously described^42^. A 1,271-bp right-flank (RF) containing the C-terminal of the *ttcA* coding region and a 1,093-bp left-flank (LF) containing the N-terminal was separately amplified from PAO1 genomic DNA using primers EBI1009 and EBI1010 and primers EBI1007 and EBI1008, respectively. The RF fragment was digested with PstI and the 1,010-bp RF fragment was isolated and cloned into pUC18::Gm^r6^ digested with HindIII/blunted and PstI yielding pUC*ttcA*R::Gm^r^. The LF fragment was digested with NcoI and the 931-bp was isolated and cloned into pUC*ttcA*R::Gm^r^ digested with MunI/blunted and NcoI yielding pUCΔ*ttcA*::Gm^r^. The constructed plasmid resulted in the deletion of 721 bp of the *ttcA* coding region. pUCΔ*ttcA*::Gm^r^ was transferred into PAO1, and the Δ*ttcA*::Gm^r^ mutants were selected for the Gm^r^ and Cb^s^ phenotypes. An unmarked Δ*ttcA* mutant was created using the Cre-*loxP* system to excise the Gm^r^ gene as previously described^42^. To construct the Δ*katA*Δ*ttcA* mutant, the pUCΔ*ttcA*::Gm^r^ was transferred into Δ*katA* mutant^36^ and followed by similar methods.

### Construction of plasmid and mini-Tn7 harbouring *ttcA*-coding regions

A pBBR-TtcA for ectopic expression of *ttcA* was constructed by amplifying the full-length *ttcA* with primers BT4673 and BT4674. The 868-bp PCR products were cloned into the medium-copy-number expression vector pBBR1MCS-4^43^ cut with SmaI, yielding pBBR-TtcA. Single-copy complementation was performed using a mini-Tn7 system^41^. The full-length *ttcA* were cut from pBBR-TtcA and cloned into pUC18-mini-Tn7T-Gm-LAC^41^ prior to transposing into either PAO1 or mutant strains, generating overexpressed (PAO1::Tn-ttcA) or complemented (Δ*ttcA*::Tn-ttcA) strains.

### Construction of Δ*oxyR* mutant and plasmid harbouring *oxyR*-coding regions

The *oxyR* deletion mutant was constructed as similar as the *ttcA* deletion mutant construction excepting with primers, BT5910 and BT5911, and a 625-bp deletion site in the *oxyR*-coding region was in between restriction enzymes, XhoI/blunted and SacII. A pBBR-OxyR for ectopic expression of *oxyR* was constructed as similar as pBBR-TtcA construction excepting with primers, EBI1047 and EBI1048.

### Construction of *katB*^−^ and ∆*ttcAkatB*^−^ mutants

The *katB* knockout mutant was constructed by an insertional inactivation method using pKNOCK vector^44^ as previously described^35^. The *katB* fragment amplified from PAO1 genomic DNA with primers, BT5639 and BT5640, was cloned into pKNOCK_Gm_ digested with SmaI, generating pKNOCK_Gm_*katB*, which was introduced into PAO1 by conjugation. The trans-conjugants were selected by the Gm^r^ phenotype. To construct a double ∆*ttcAkatB*^−^ mutant, the pKNOCK_Gm_*katB* was introduced into the genome of ∆*ttcA* mutant. The mutants were confirmed by PCR and DNA sequencing.

### Site-directed mutagenesis of TtcA

Site-directed mutagenesis was performed to convert cysteine residues (C38, C115, C118, C184, C203, or C206) to serine residues through PCR-based mutagenesis as previously described^6^. To construct pTn-*ttcA*C38S for the expression of TtcA-C38S, two pairs of primers EBI1011-TN7S and EBI1012-BT5250, were used in two-step PCR using pUC18-mini-Tn7T-Gm-*ttcA* as a template. The PCR product was digested with EcoRI and SacI prior to cloning into pUC18-mini-Tn7T-Gm-LAC, generating pTn-*ttcA*C38S. pTn-*ttcA*C115S, pTn-*ttcA*C118S, pTn-*ttcA*C184S, pTn-*ttcA*C203S and pTn-*ttcA*C206S were constructed using the same protocol with different sets of mutagenic primers: EBI1013 and EBI1014 for C115S, EBI1015 and EBI1016 for C118S, EBI1017 and EBI1018 for C184S, EBI1019 and EBI1020 for C203S, and EBI1021 and EBI1022 for C206S. The presence of each mutation was verified by DNA sequencing.

### Expression and purification of *P*. *aeruginosa* TtcA

6His-tagged TtcA from *P*. *aeruginosa* was expressed using the pQE-30Xa expression system (Qiagen, Germany) under oxygen-limited conditions as previously described^34^. The full-length *ttcA* gene was amplified from PAO1 genomic DNA with the primers EBI1035 and EBI1036. An 835-bp PCR product was digested with HindIII before ligation into pQE-30Xa digested with StuI/blunted and HindIII to generate pQE-30Xa-*ttcA* for the high-level expression of *ttcA* containing an N-terminal 6His-tag. An *E*. *coli* M15 strain harbouring pQE-30Xa-*ttcA* was grown to an OD_600_ of 1.0 before being induced with 100 μM IPTG for 60 min with 40 rpm shaking. Purification of 6His-tagged TtcA was carried out using a nickel-nitrilotriacetic acid (Ni-NTA) agarose column as previously described^34^. The purity of the TtcA protein was more than 95%, as judged by a major band corresponding to the 32.3-kDa protein observed on SDS-PAGE. UV-Visible spectrophotometry was used to analyse the relative amount of [Fe-S] cluster using the ratio of absorbance 420 nm and 280 nm.

### Plate sensitivity assay

A plate sensitivity assay was performed to determine the oxidant resistance level as previously described^7^. Briefly, exponential phase cells were adjusted to OD_600_ of 0.1 before making 10-fold serial dilutions. 10 μl of each dilution was then spotted onto LB agar plate containing testing reagents. The plates were incubated overnight at 37 °C before the colony forming units (CFU) were scored. Percent survival was defined as the percentage of the CFU on plates containing oxidant divided by the CFU on plates without oxidant.

### Hydrogen peroxide, NaOCl, heat and pH susceptibility test

A susceptibility assay was performed to determine the stress resistance level as previously described^35^. In short, exponential-phase cultures were normalized to an OD_600_ of 0.1 before treating with lethal concentrations of either H_2_O_2_, NaOCl, heat, acidic pH, or basic pH for 30 min at 37 °C. After treatment, cells were immediately washed twice with fresh LB broth. Cells that survived the treatment were scored using a viable cell count. The resistance levels against these stresses were expressed as the % survival, defined as the percentage of the CFU with treatment divided by the CFU without treatment.

### Catalase activity assays

Total catalase activity in *P*. *aeruginosa* cells was measured by spectrophotometrically monitoring the decomposition of hydrogen peroxide^6^. Briefly, the reaction was performed by mixing bacterial lysate with 30 mM H_2_O_2_ in 50 mM phosphate buffer pH 7.0. The absorbance changes at A_240_ were recorded at time intervals and calculated as the specific activity of catalase (U mg^−1^ protein). One unit of catalase was defined as the amount of enzyme required to hydrolyse 1 µmol of H_2_O_2_ per min at 25 °C, pH 7.0, and the molar extinction e_240_ was equal to 0.041 cm^2^ µmol^−1^.

The gel activity of Kat was intensely measured from native PAGE of *P*. *aeruginosa* cell extracts, which were stained for Kat activity as previously described^45^. Thirty milligrams (unheated) of protein were loaded, and protein concentrations were estimated using Bradford assay (Bio-Rad, USA). The stained gel was renatured, and catalase activity was visualized following a previously described method^46^ with some modifications by washing twice before soaking with horseradish peroxidase (Sigma, USA) and then removing this enzyme. The gel was immediately soaked in 5 mM H_2_O_2_ and stained with 3,3′diaminobenzidine. Catalase activity was visualized as colourless bands against a brownish background.

### Western blot analysis

Western blot analysis was performed as previously described^47^. In brief, crude protein was extracted and isolated before mixing with 6X protein loading dye and boiling for 10 min. The stained protein was run under 12.5% SDS-PAGE and transferred to a Hybond PVDF membrane (GE Healthcare) in a semi-dry transfer cell (Bio-Rad). The transferred membrane was blocked and hybridized with anti-6His-peroxidase primary antibody (Roche, Switzerland) and developed with Ultra TMB-Blotting Solution (Thermo Scientific) according to the manufacturer’s recommendation.

### *Drosophila* virulence test

The virulence of *P*. *aeruginosa* was investigated using the *Drosophila melanogaster* feeding assay as previously described^7^. Shortly, 800 µL of *P*. *aeruginosa* cultures were overlaid to completely cover the surface of the corn flour *Drosophila* medium in a glass fly culture vial. One-week-old adult flies were starved for 3 hours prior to the feeding assay. Twenty flies were added to each vial and incubated at 25 °C before the number of the viable flies was observed at different time points. The experiments were performed in a double-blind fashion and were analyzed from nine experiments using three different batches of flies.

### Real time RT-PCR

RNA extraction and reverse transcription was performed as previously mentioned^6,35^. Real time RT-PCR was conducted using a SYBR^®^ FAST qPCR kit (KAPA Biosystems, USA). The reaction was run on an Applied Biosystems StepOnePlus thermal cycler under the recommended fast protocol condition. The specific primer pairs used for *ttcA*, *katA*, *katB*, *oxyR*, and *tpx* were BT4675-BT4676, BT5637-BT5638, BT5639-BT5640, EBI163-EBI164 and BT3186-BT3787, respectively^35^. The primer pair for the 16 S rRNA gene was BT2781-BT2782, which was used as the normalizing gene. Relative expression analysis was calculated using StepOne software and is presented as expression fold-change relative to the level of uninduced conditions. Data shown are the means with standard deviations (SD) from three biologically independent experiments.

### 5′ rapid amplification of cDNA ends (RACE)

5′ RACE was performed using a 5′/3′ RACE kit (Roche, Switzerland) as previously described^34^. Essentially, DNase I-treated total RNA was reverse transcribed using specific primers EBI341 as SP1 primers. The first-strand DNA (cDNA) was purified, and poly(A) was added to the 5′-terminus of the cDNA using terminal transferase. Next, poly(A)-tailed cDNA was PCR-amplified using the specific SP2 primer BT4991 and an anchored oligo(dT) primer. The purified PCR product was cloned into the pGEM-T Easy vector, and the +1 site was identified from the DNA sequences.

### OxyR purification and electrophoresis mobility shift assay

6His-tagged OxyR from *P*. *aeruginosa* was purified using the pQE-30Xa expression system in a manner similar to that used for TtcA purification. The purity of OxyR was more than 90% as a 35 kDa major band observed in SDS-PAGE. EMSA was performed as previously described^34^ using a ^32^P-labeled probe containing the putative *ttcA* promoter. The probe was amplified with ^32^P-labeled BT4990 and BT4991 primers. Binding reactions consisting of labeled probe in 20 µl of reaction buffer containing 20 mM Tris-HCl (pH 7.0), 50 mM KCl, 1 mM EDTA, 5% glycerol, 50 μg ml^−1^ BSA, 5 μg ml^−1^ calf thymus DNA, 0.5 μg ml^−1^ poly[dI-dC], 50 μg ml^−1^ salmon sperm DNA, 100 mM DTT, and purified OxyR were incubated at 25 °C for 30 min. Gel electrophoresis and visualization was done as previously described^34^.

### Statistics

Group data are presented as means ± standard deviation (SD). The Student’s t-test was used to determine differences between means using the function of Excel (Microsoft, Washington) and the SPSS (version 17.0; SPSS Inc.) statistical package. Unless otherwise is stated, *p* values of <0.05 were considered significant.

### Ethics statement

All *P*. *aeruginosa* and *D*. *melanogaster* were raised, maintained and all experiments were conducted following procedures, MUSC2016-002 and MUSC60-039-389, approved by the Committee of Biosafety, Faculty of Science, Mahidol University (MUSC) and the MUSC-Institutional Animal Care and Use Committee (IACUC), respectively.

## Electronic supplementary material

Supplementary Fig1–4 Table1–2
