# Supplementary material for: Pseudomonas aeruginosa ttcA encoding tRNA-thiolating protein requires an iron-sulfur cluster to participate in hydrogen peroxide-mediated stress protection and pathogenicity
Source: Sci Rep. 2018 Aug 8;8:11882. doi: 10.1038/s41598-018-30368-y (PMC6082896; doi:10.1038/s41598-018-30368-y)
Supplement: Supplementary file 1 — Supplementary Fig1–4 Table1–2 [file 41598_2018_30368_MOESM1_ESM.pdf]

Supplementary Information

Pseudomonas aeruginosa ttcA encoding tRNA-thiolating protein requires an iron-sulfur cluster to participate in hydrogen peroxide-mediated stress protection and pathogenicity

Adisak Romsang*, Jintana Duang-nkern, Khwannarin Khemsom, Lampet Wongsaroj, Kritsakorn Saninjuk, Mayuree Fuangthong, Paiboon Vattanaviboon, and Skorn Mongkolsuk

1 2 3 4 5 6 7 8

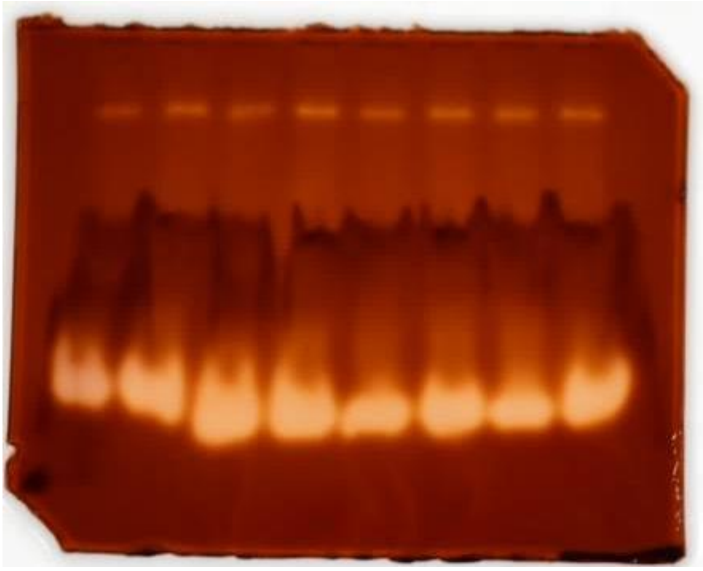

1 PAO1::Tn
2 PAO1::Tn-TtcA
3 PAO1::Tn
4 PAO1::Tn-TtcA
5 Δ*ttcA*::Tn
6 Δ*ttcA*::Tn-TtcA
7 Δ*ttcA*::Tn
8 Δ*ttcA*::Tn-TtcA

Supplementary Fig S1. Original full image of Figure 4B showing KatA and KatB catalase gel activities were investigated among *P. aeruginosa* strains as an indicated number of each lane. Two biologically independent clones of each strain were used in the experiment of catalase gel activity detection and data analysis.

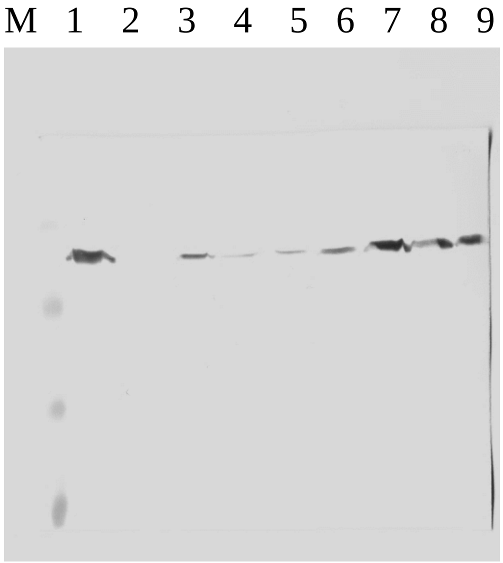

M Protein marker
1 PAO1/p*katA*-6His
2 Δ*katA*::Tn/pBBR
3 Δ*katA*::Tn/p*katA*-6His
4 Δ*katA*Δ*ttcA*::Tn/p*katA*-6His
5 Δ*katA*Δ*ttcA*::TtcA/p*katA*-6His
6 Δ*katA*::Tn/p*katA*-6His
7 Δ*katA*::Tn/p*katA*-6His (10X)
8 Δ*katA*Δ*ttcA*::Tn/p*katA*-6His (10X)
9 Δ*katA*Δ*ttcA*::Tn-TtcA/p*katA*-6His (10X)

Supplementary Fig S2. Original full image of Figure 5B showing Western blot analysis of 6His-KatA levels in *P. aeruginosa* strains determined using a mouse anti-6His antibody was presented as an indicated number of each lane. Crude proteins were prepared from an equal amount of *P. aeruginosa* culture, and electrophoresis was carried out using 12.5% SDS–PAGE with protein markers. Ten times volume of protein indicated as 10X was used as a repeat during analysis.

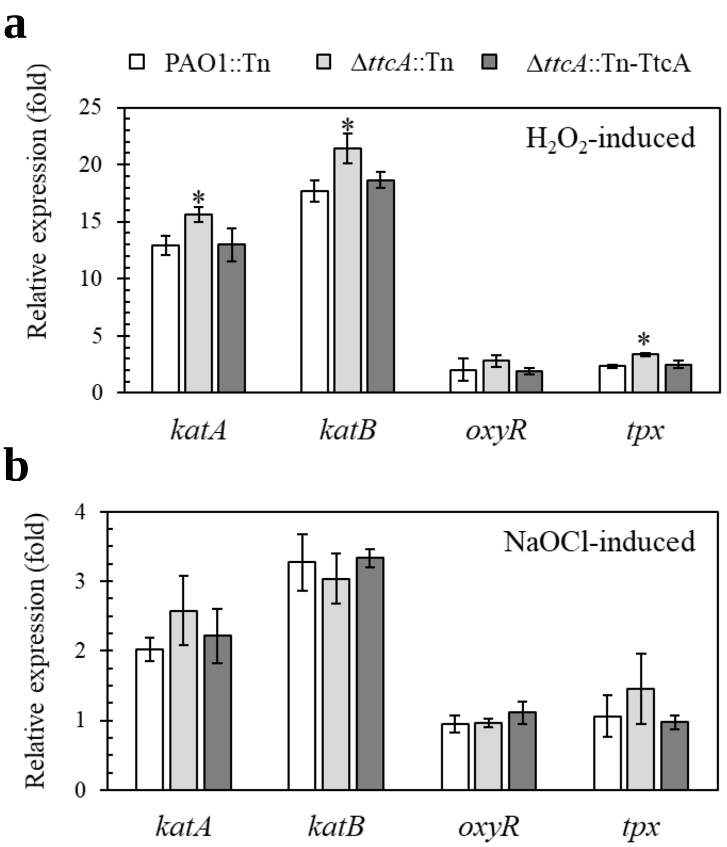

Supplementary Fig S3. Expression levels of *katA*, *katB*, *oxyR*, and *tpx* in wild-type PAO1 and Δ*ttcA* mutants containing either Tn or Tn-TtcA insertion were determined using real time RT-PCR. Bacterial cultures were subjected to either (A) 0.25 mM H_2O_2 or (B) 0.002% NaOCl for 15 minutes prior to RNA extraction. Relative expression was analyzed using the 16S rRNA gene as the normalizing gene and was expressed as the fold expression relative to the level of the PAO1 without treatment. The data shown are means and SD from three biologically independent experiments. The asterisks indicate statistically significant differences ($p < 0.01$) compared with the level of the PAO1 without treatment.

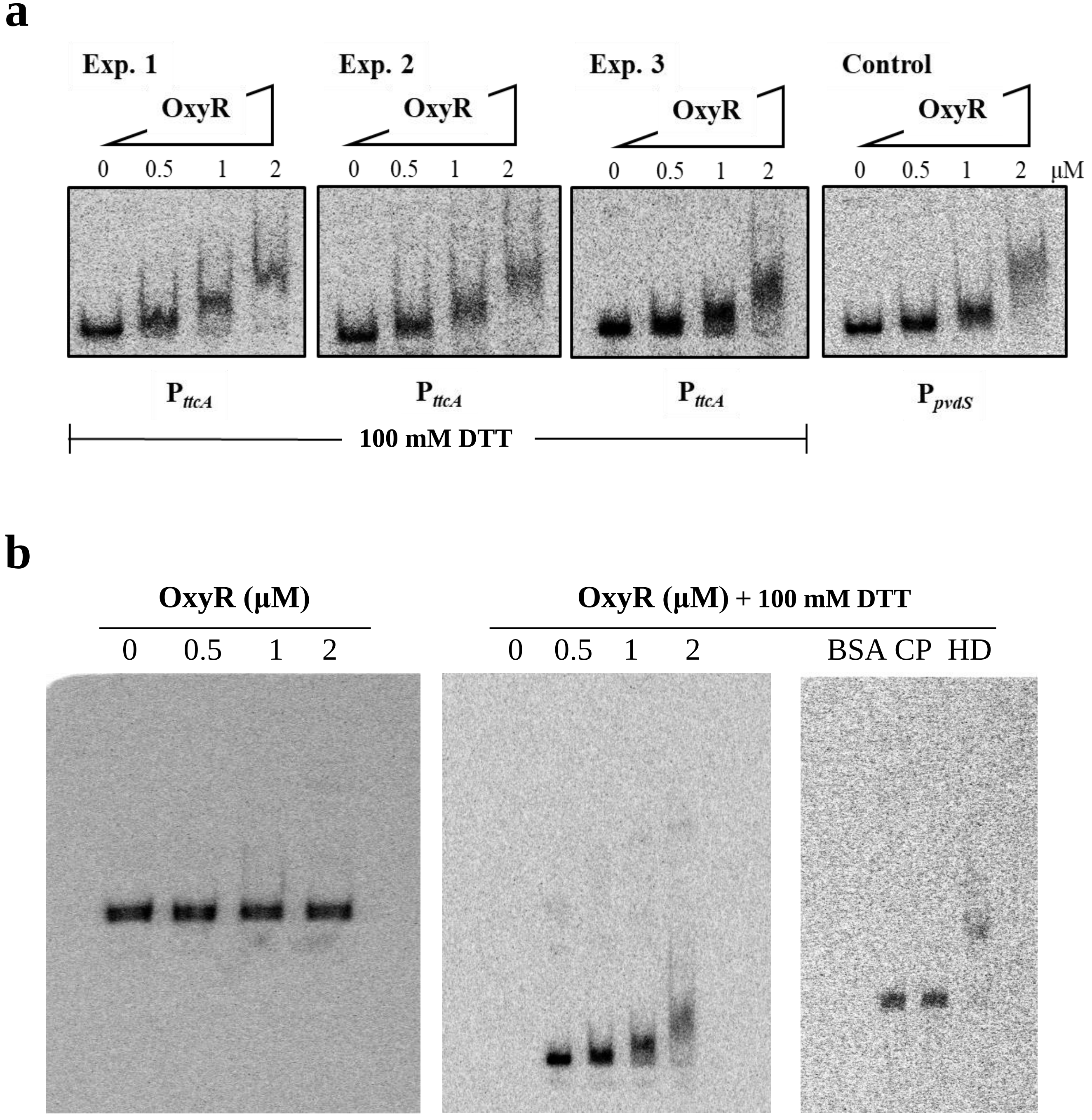

Supplementary Fig S4. (A) Electrophoretic mobility shift assay of OxyR-binding *ttcA* promoter from three independent experiments and EMSA of OxyR-binding *pvdS* promoter as a known target control (B) Original full images presented in Figure 8A determined by EMSA using ^{32}P-labeled *ttcA* promoter fragment and increasing concentrations of purified OxyR treated with 100 mM DTT.

Supplementary Table S1. List of plasmids used in this study.

Plasmid	Relevant characteristic(s)	Source or Reference
pBBR1MCS-4	Broad-host-range expression vector, Ap^r	Kovach ME *et al*, 1995
pBBR-TtcA	pBBR1MCS-4 containing *ttcA*, Ap^r	This study
pBBR-OxyR	pBBR1MCS-4 containing *oxyR*, Ap^r	This study
$pKnock_{GM}$	Suicide vector, Gm^r	Alexeyev MF, 1999
$pKnock_{GM}$-KatB	$pKnock_{GM}$ containing a partial *katB* encoding region, Gm^r	This study
pBBR-OxyR	pBBR1MCS-4 containing *oxyR*, Ap^r	This study
pUCΔ*ttcA*::Gm^r	pUC18 containing Gm^r inserted into deleted *ttcA*, Gm^r	This study
pUCΔ*oxyR*::Gm^r	pUC18 containing Gm^r inserted into deleted *oxyR*, Gm^r	This study
pCM351	vector containing the *loxP*-Gm^r-*loxP* region, Gm^r	Marx CJ *et al*, 2002
pCM157	vector containing the Cre-encoding gene, Tet^r	Marx CJ *et al*, 2002
pUC18-mini-Tn7T::Gm-LAC	mini-Tn7 vector with P_{lac} expression cassette, Gm^r	Choi KH *et al*, 2006
pTNS2	Helper plasmid for Tn7 insertion, Ap^r	Choi KH *et al*, 2006
pTn-TtcA	pUC18-mini-Tn7T::Gm-LAC containing *ttcA*, Gm^r	This study
pTn-TtcA C38S	pUC18-mini-Tn7T::Gm-LAC containing mutagenized *ttcA* C38S, Gm^r	This study
pTn-TtcA C115S	pUC18-mini-Tn7T::Gm-LAC containing mutagenized *ttcA* C115S, Gm^r	This study
pTn-TtcA C118S	pUC18-mini-Tn7T::Gm-LAC containing mutagenized *ttcA* C118S, Gm^r	This study
pTn-TtcA C184S	pUC18-mini-Tn7T::Gm-LAC containing mutagenized *ttcA* C184S, Gm^r	This study
pTn-TtcA C203S	pUC18-mini-Tn7T::Gm-LAC containing mutagenized *ttcA* C203S, Gm^r	This study
pTn-TtcA C206S	pUC18-mini-Tn7T::Gm-LAC containing mutagenized *ttcA* C206S, Gm^r	This study
pQE30Xa	Vector for expressing N-terminal 6His tagged protein in *E. coli*, Ap^r, Cm^r	Qiagen (Germany)
pQE30Xa-*ttcA*	pQE30Xa carrying full-length *ttcA*, Ap^r	This study
pQE30Xa-*oxyR*	pQE30Xa carrying full-length *oxR*, Ap^r	This study

Supplementary Table S2. List of primers used in this study.

Name	Sequence 5'→3'	Purpose
BT2781	GCCCGCACAAGCGGTGGAG	Forward primer for 16S rRNA
BT2782	ACGTCATCCCCACCTTCCT	Reverse primer for 16S rRNA
BT3186	TATCTCCGAACGCCAAGG	Forward primer for *tpx*
BT3187	GGTGGTGGGTCAGACAGG	Reverse primer for *tpx*
BT4673	GATTCCAGAGCACCATGGG	Forward primer for full-length *ttcA*
BT4674	CCGGAGAGAAGAACTGACC	Reverse primer for full-length *ttcA*
BT4675	GTGAACATGGACCAGAAGCG	Forward primer for *ttcA* expression
BT4676	GTCTCGAGGATGTCGTCG	Reverse primer for *ttcA* expression
BT4990	AGGTCGAGGCGGTGGTCG	Forward primer for *ttcA* promoter
BT4991	AAGTCGGTGATGGCCTCG	Reverse primer for *ttcA* promoter and Sp2 primer
BT5250	ATGTGTGGAATTGTGAGCA	Forward primer in pUC18-mini-Tn7T::Gm-LAC
BT5637	TCTCCATGCGTTTCTACACC	Forward primer for *katA* expression
BT5638	CGCATTGATGAAGCTGAAGG	Reverse primer for *katA* expression
BT5639	CGACGCTTCGATTTCTTCTC	Forward primer for *katB* expression and knockout
BT5640	TTCGGATCGAGGTTCTTCTG	Reverse primer for *katB* expression and knockout
BT5910	CGGTGCTGGAGACGAACAG	Forward primer for upstream fragment of *oxyR*
BT5911	CTTCGGTAGTCGGGTAGATC	Reverse primer for downstream fragment of *oxyR*
EBI163	TCGGCGCCATCTACACCATC	Forward primer for *oxyR* expression
EBI164	GCAGGCTCTTGTCGTTGAG	Reverse primer for *oxyR* expression
EBI341	GCAGGATGTCGAGCATGG	Sp1 primer of *ttcA*
EBI1007	GATGCTTTCTTCTTCGCC	Forward primer for upstream fragment of *ttcA*
EBI1008	TAGAGCAGGATGTCGAGC	Reverse primer for upstream fragment of *ttcA*
EBI1009	CAACATGTTCTACGGCGG	Forward primer for downstream fragment of *ttcA*
EBI1010	GGCTGAAATACCTGCTGC	Reverse primer for downstream fragment of *ttcA*
EBI1011	GTCATGGTCTGCCTGTCCGGC	Forward primer for site-directed mutagenesis C38S
EBI1012	GCCGGACAGGCAGACCATGAC	Reverse primer for site-directed mutagenesis C38S
EBI1013	AAGACCACCTGCTCGCTGTGC	Forward primer for site-directed mutagenesis C115S
EBI1014	GCACAGCGAGCAGGTGGTCTT	Reverse primer for site-directed mutagenesis C115S
EBI1015	TGCTCGCTGTGCTCGCGCCTG	Forward primer for site-directed mutagenesis C118S
EBI1016	CAGGCGCGAGCACAGCGAGCA	Reverse primer for site-directed mutagenesis C118S
EBI1017	CTGGCCTATTGCAGCGAGAAG	Forward primer for site-directed mutagenesis C184S
EBI1018	CTTCTCGCTGCAATAGGCCAG	Reverse primer for site-directed mutagenesis C184S
EBI1019	ATCATCCCCTGCAACCTCTGC	Forward primer for site-directed mutagenesis C203S
EBI1020	GCAGAGGTTGCAGGGGATGAT	Reverse primer for site-directed mutagenesis C203S
EBI1021	TGCAACCTCTGCGGTTCGCAG	Forward primer for site-directed mutagenesis C206S
EBI1022	CTGCGAACCGCAGAGGTTGCA	Reverse primer for site-directed mutagenesis C206S
EBI1035	ATGGGCACCCTTTCGGTCAATCAG	Forward primer for TtcA protein expression
EBI1036	GCGGAAGCTTTCAGAGGTTCATCACGTC	Reverse primer for TtcA protein expression
EBI1047	GATCTTAACGGATGAGCAGC	Forward primer for full-length *oxyR*
EBI1048	AGCTCGGTCATGCTATTTGC	Reverse primer for full-length *oxyR*
TN7S	GATGGGAACTGGGTGTAGCG	Reverse primer in pUC18-mini-Tn7T::Gm-LAC
